# Homocysteine-induced sustained GluN2A NMDA receptor stimulation leads to mitochondrial ROS generation and neurotoxicity

**DOI:** 10.1016/j.jbc.2024.107253

**Published:** 2024-04-01

**Authors:** Satya Narayan Deep, Sarah Seelig, Surojit Paul, Ranjana Poddar

**Affiliations:** Department of Neurology, University of New Mexico Health Sciences Center, Albuquerque, New Mexico, USA

**Keywords:** homocysteine, GluN2A-NMDA receptor, Pyk2, Src family kinase, Ca^2+^ influx, mitochondrial reactive oxygen species, ERK MAPK, neurotoxicity

## Abstract

Homocysteine, a sulfur-containing amino acid derived from methionine metabolism, is a known agonist of N-methyl-D-aspartate receptor (NMDAR) and is involved in neurotoxicity. Our previous findings showed that neuronal exposure to elevated homocysteine levels leads to sustained low-level increase in intracellular Ca^2+^, which is dependent on GluN2A subunit-containing NMDAR (GluN2A-NMDAR) stimulation. These studies further showed a role of ERK MAPK in homocysteine-GluN2A-NMDAR–mediated neuronal death. However, the intracellular mechanisms associated with such sustained GluN2A-NMDAR stimulation and subsequent Ca^2+^ influx have remained unexplored. Using live-cell imaging with Fluo3-AM and biochemical approaches, we show that homocysteine-GluN2A NMDAR-induced initial Ca^2+^ influx triggers sequential phosphorylation and subsequent activation of the proline rich tyrosine kinase 2 (Pyk2) and Src family kinases, which in turn phosphorylates GluN2A-Tyr^1325^ residue of GluN2A-NMDARs to maintain channel activity. The continuity of this cycle of events leads to sustained Ca^2+^ influx through GluN2A-NMDAR. Our findings also show that lack of activation of the regulatory tyrosine phosphatase STEP, which can limit Pyk2 and Src family kinase activity further contributes to the maintenance of this cycle. Additional studies using live-cell imaging of neurons expressing a redox-sensitive GFP targeted to the mitochondrial matrix show that treatment with homocysteine leads to a progressive increase in mitochondrial reactive oxygen species generation, which is dependent on GluN2A-NMDAR–mediated sustained ERK MAPK activation. This later finding demonstrates a novel role of GluN2A-NMDAR in homocysteine-induced mitochondrial ROS generation and highlights the role of ERK MAPK as the intermediary signaling pathway between GluN2A-NMDAR stimulation and mitochondrial reactive oxygen species generation.

The N-methyl-D-aspartate subtype of glutamate receptors (NMDARs) plays a critical role in promoting brain development, learning, and memory as well as in mediating the pathology of multiple neurological disorders. Earlier studies have shown that this opposing role of NMDARs is dependent on the subunit composition of the functional NMDAR complex at the neuronal surface. In the adult brain, functional NMDARs are predominantly composed of heteromeric assemblies of GluN1/GluN2A, GluN1/GluN2B, or GluN1/GluN2A/GluN2B subunits (1, 2, 3, 4, 5). Stimulation of GluN2A subunit containing NMDARs (GluN2A-NMDAR) by the NMDAR agonist and neurotransmitter glutamate leads to moderate and transient influx of Ca^2+^, resulting in activation of intracellular signaling pathways that promote synaptic plasticity, neuronal survival, and growth (2, 4, 6, 7, 8, 9), whereas stimulation of GluN2B subunit containing NMDARs (GluN2B-NMDAR) following excessive glutamate release results in large Ca^2+^ influx in neurons that leads to activation of several detrimental pathways and neuronal death, a phenomenon known as excitotoxicity (4, 9, 10, 11, 12). GluN2B-NMDAR–mediated excitotoxic cell death has been shown to be involved in the progression of multiple neurological disorders, including cerebral ischemia, traumatic brain injury, epilepsy, multiple sclerosis, and amyotrophic lateral sclerosis.

Homocysteine, a thiol-containing amino acid formed as an intermediate of the methionine cycle is another NMDAR agonist (13), and systemic elevation of plasma homocysteine also known as hyperhomocysteinemia is a metabolic disorder that is recognized as an independent risk factor for both acute and chronic neurological disorders (14, 15, 16, 17, 18, 19). In contrast to glutamate-mediated excitotoxicity, homocysteine induces neuronal cell death through a novel signaling pathway that involves GluN2A-NMDAR stimulation (13, 20, 21, 22). Studies in neuronal cultures have shown that homocysteine-mediated stimulation of GluN2A-NMDARs, but not GluN2B-NMDARs, leads to continuous and progressive increase in intracellular Ca^2+^, resulting in sustained increase in the extracellular signal-regulated kinase, a member of the mitogen activated protein kinase family (ERK MAPK) phosphorylation, which eventually leads to neuronal cell death (23). Using a rodent model of cerebral ischemic stroke, it has also been shown that predisposition to hyperhomocysteinemia exacerbates ischemic brain injury, which involves GluN2A-NMDAR–mediated sustained increase in ERK MAPK phosphorylation (24). Collectively, these findings suggest that sustained GluN2A-NMDAR stimulation plays a crucial role in homocysteine-mediated neuronal cell death. However, the precise molecular mechanism(s) underlying such unregulated GluN2A-NMDAR stimulation following exposure to elevated levels of homocysteine is not known. Furthermore, the signaling events downstream of GluN2A-NMDAR that are involved in neuronal cell death are also not well understood. In this context, although changes in cellular redox status are often linked to hyperhomocysteinemia-associated neurodegenerative disorders (25, 26), the causal link between homocysteine-induced GluN2A-NMDAR stimulation and the increase in intracellular oxidative stress is still not clear.

A significant goal of the current study is to unravel whether homocysteine-induced GluN2A-NMDARs activation is a critical determinant of mitochondrial reactive oxygen species (ROS) generation. To address this issue, we investigated whether homocysteine-GluN2A-NMDAR mediated initial Ca^2+^ influx and Ca^2+^-dependent ERK MAPK phosphorylation triggers a feedforward cycle to enhance the duration of GluN2A-NMDAR stimulation and ERK MAPK phosphorylation. Subsequent studies further examined whether such sustained increase in ERK MAPK phosphorylation plays a key role in mitochondrial ROS generation that results in neurotoxicity.

## Results

### Homocysteine-mediated phosphorylation of Pyk2 kinase and SFKs leads to sustained influx of Ca^2+^ through GluN2A-NMDAR in neurons

**Figure 1:**
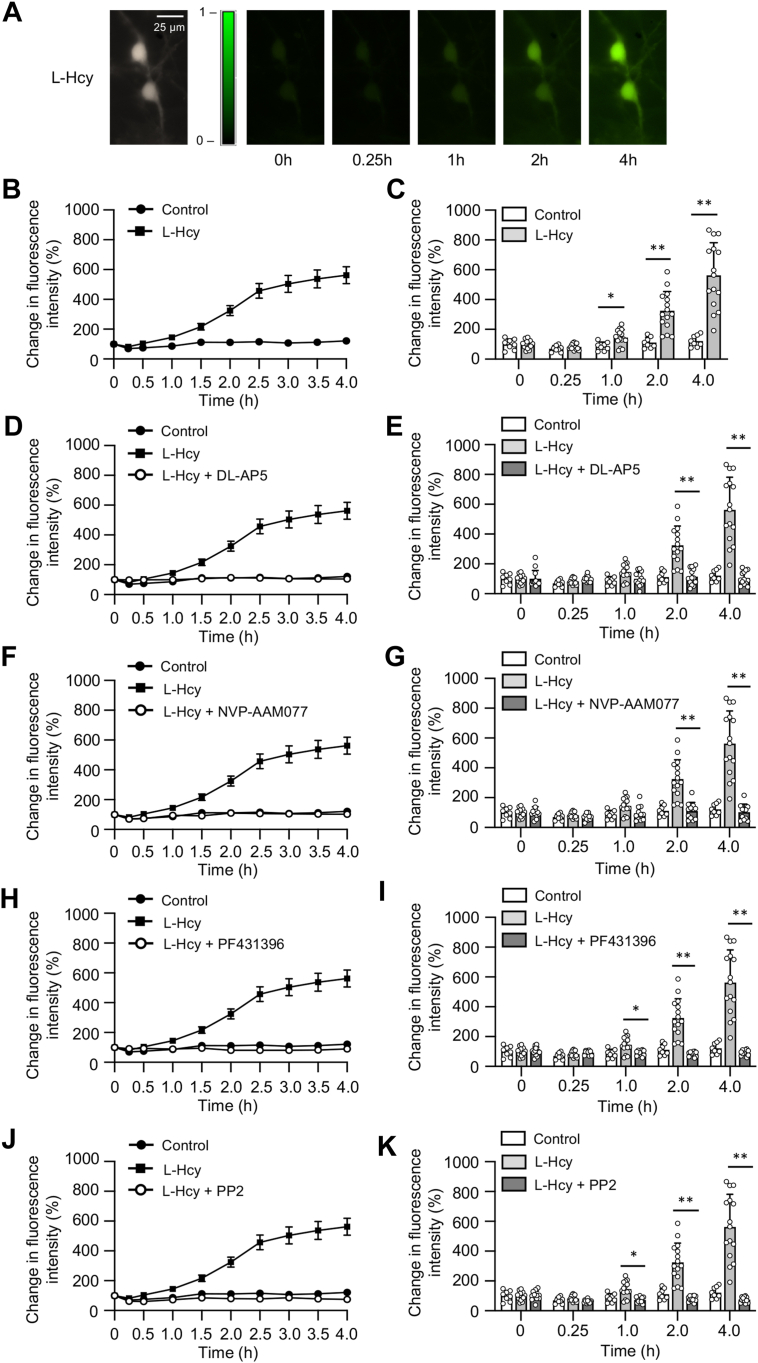
**Role of Pyk2 and SFK in homocysteine-induced changes in intracellular Ca**^**2+**^**in neurons.***A*, representative micrographs of Fluo3-AM loaded neurons showing changes in intracellular Ca^2+^ over time measured at 484 nm, following exposure to L-homocysteine (50 μM). Both *black* and *white* (4 h) *and false-color images* (0–4 h) are shown. *B*, temporal profile of intracellular Ca^2+^ increase over time was assessed by measuring increase in Fluo3-AM fluorescence intensity (mean ± SE) in soma of L-Hcy–treated and L-Hcy–untreated (control) neurons. *C*, individual responses in neuronal soma showing the range of increase in intracellular Ca^2+^ at specific time points, following exposure to L-Hcy, expressed as mean ± SD. Two-way ANOVA followed by Bonferroni’s multiple-comparisons test shows significant effect (treatment effect: F (_1, 21_) = 33.42; *p* < 0.0001, time effect: F _(1.629, 34.21)_ = 32.49; *p* < 0.0001, interaction: F (_4, 84_) = 23.09; *p* < 0.0001). *D*, *F*, *H*, and *J*, temporal profile of changes in intracellular Ca^2+^ in neurons treated with L-Hcy (50 μM) in the presence or absence of (*D*) NMDAR inhibitor DL-AP5 (200 μM); (*F*) GluN2A-NMDAR inhibitor NVP-AAM077 (30 nM); (*H*) Pyk2 inhibitor PF431396 (5 μM); or (*J*) SFK inhibitor PP2 (5 μM) expressed as mean ± SE. *E*, *G*, *I*, and *K*, quantitative analysis of individual response in neuronal soma showing the range of increase in intracellular Ca^2+^ at the specified time points, following treatment with L-Hcy and in the presence or absence of pharmacological inhibitors expressed as mean ± SD. Two-way ANOVA followed by Bonferroni’s multiple-comparisons test shows significant effect of treatment, time, and interaction in the presence of (*E*) NMDAR inhibitor DL-AP5 (treatment effect: F _(2, 33)_ = 36.83; *p* < 0.0001, time effect: F _(1.803, 59.50)_ = 33.62; *p* < 0.0001, interaction: F _(8, 132)_ = 29.75; *p* < 0.0001), (*G*) GluN2A-NMDAR inhibitor NVP-AAM077 (treatment effect: F _(2, 31)_ = 33.16; *p* < 0.0001, time effect: F _(1.701, 52.74)_ = 33.69; *p* < 0.0001, interaction: F _(8, 124)_ = 27.59; *p* < 0.0001), (*I*) Pyk2 inhibitor PF431396 (treatment effect: F _(2, 32)_ = 43.50; *p* < 0.0001, time effect: F _(1.662, 53.18)_ = 32.93; *p* < 0.0001, interaction: F _(8, 128)_ = 32.33; *p* < 0.0001), and (*K*) SFK inhibitor PP2 (treatment effect: F _(2, 32)_ = 47.81; *p* < 0.0001, time effect: F _(1.663, 53.21)_ = 33.83; *p* < 0.0001, interaction: F _(8, 128)_ = 32.13; *p* < 0.0001). Post hoc analysis shows ∗*p* < 0.01 and ∗∗*p* < 0.0001 between the treatment groups at the given time points. Values are mean ± SD. Data points represent individual biological replicates. NMDAR, N-methyl-D-aspartate subtype of glutamate receptor; SFK, Src family kinase.

In an earlier study, we demonstrated that the treatment of neuronal cultures with homocysteine causes GluN2A-NMDAR stimulation (23). Using the high affinity Ca^2+^ dye Fura2-AM, the study further showed that homocysteine-induced GluN2A-NMDAR stimulation leads to a slow and progressive increase in Ca^2+^ influx over a period of 1 h. Although Fura-2 AM allows accurate quantification of intracellular Ca^2+^ level, it has a short half-life (27, 28, 29), thereby limiting its use for long-term evaluation of intracellular Ca^2+^. Therefore, in the current study, we used the single-wavelength Ca^2+^ indicator dye Fluo-3 AM to measure homocysteine-GluN2A-NMDAR–mediated long-term changes in intracellular Ca^2+^ dynamics monitored over a period of 4 h. Rat neuronal cultures were exposed to L-homocysteine (50 μM, 4 h) and changes in Fluo-3 AM fluorescent intensity in the cell soma was measured by live-cell imaging. The photomicrographs presented in Figure 1*A* exhibit large increase in fluorescent intensity over time on binding of the dye to intracellular Ca^2+^ (in false-color maps), following treatment with homocysteine. The line graph presented in Figure 1*B* shows the temporal profile of increase in fluorescent intensity, as an indicator of increasing intracellular Ca^2+^ levels in homocysteine treated cells when compared to vehicle-treated control cells. The corresponding bar graph at selective time points (15 min, 1 h, 2 h, and 4 h) representing Ca^2+^ changes in the soma of individual neurons shows significant increase in intracellular Ca^2+^ levels within 1 h of homocysteine treatment compared to time-matched vehicle-treated control (Fig. 1*C*). In subsequent studies, Fluo-3 AM fluorescent intensity was measured in neurons that were treated with homocysteine in the presence of NMDAR inhibitor DL-AP5 (200 μM) or selective GluN2A-NMDAR inhibitor NVP-AAM077 (30 nM). The findings (Fig. 1, *D*–*G*) show that pharmacological inhibition with either DL-AP5 or NVP-AAM077 blocks homocysteine-induced increase in intracellular Ca^2+^ levels, thus confirming the role of GluN2A-NMDAR in homocysteine-mediated sustained influx of Ca^2+^. Several lines of evidence indicate that Pyk2 and Src family kinases (SFKs) are part of the NMDAR complex and play a role in increasing surface expression and function of the receptor (30, 31, 32, 33, 34, 35, 36). Therefore, in additional studies, neurons were treated with L-homocysteine in the presence of Pyk2 inhibitor PF431396 (5 μM) or SFK inhibitor PP2 (5 μM), and Fluo3-AM fluorescence intensity was monitored for a period of 4 h. The results (Fig. 1, *H*–*K*) show that treatment with either PF431396 or PP2 blocks homocysteine-GluN2A-NMDAR–mediated increase in Fluo3-AM fluorescence intensity, indicating that homocysteine-GluN2A-NMDAR–mediated sustained influx of Ca^2+^ is dependent on the activation of Pyk2 and SFKs.

**Figure 2:**
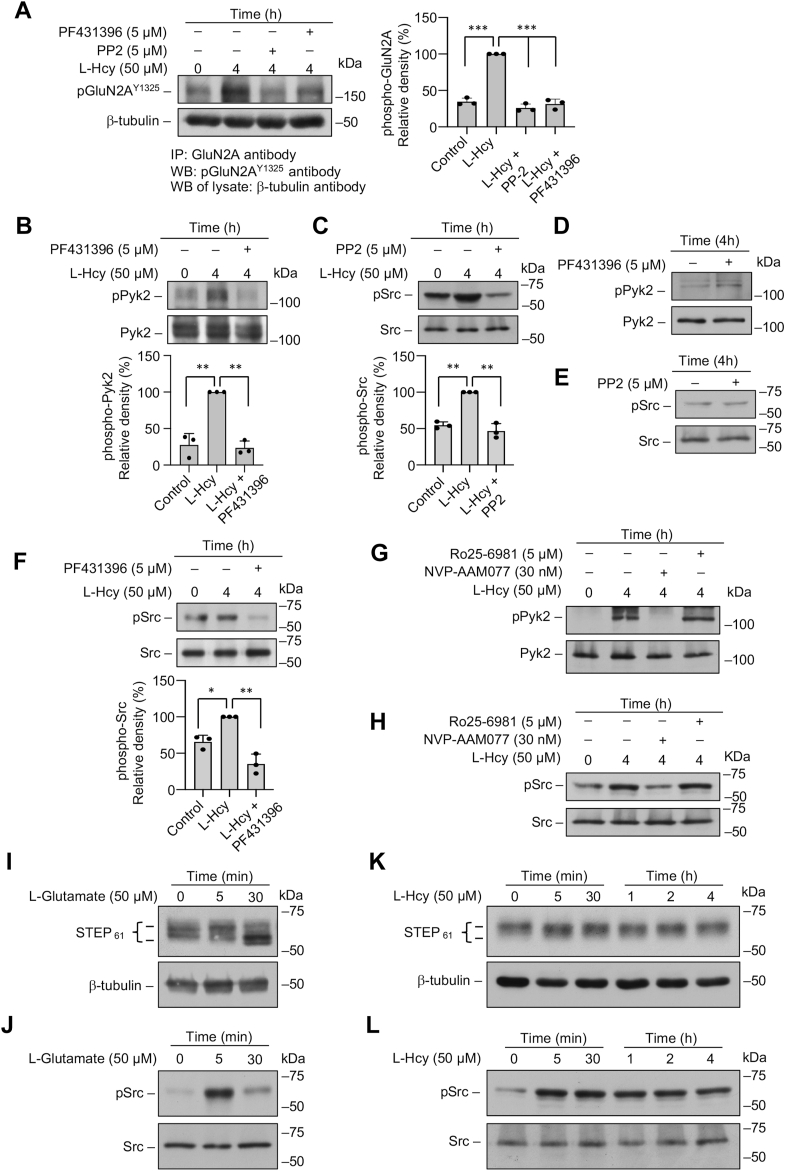
**Homocysteine mediated increase in Pyk2 and SFK phosphorylation leads to GluN2A-NMDAR phosphorylation in neurons.***A*–*F*, rat neuron cultures were treated with 50 μM L-homocysteine (L-Hcy, 4 h) in the presence and absence of Pyk2 inhibitor PF431396 (5 μM) or SFK inhibitor PP2 (5 μM). *A*, GluN2A-subunit of NMDAR was immunoprecipitated from total cell lysate using anti-GluN2A antibody, and the immune complexes were processed for immunoblot analysis using anti-pGluN2A^Y1325^ antibody (*upper panel*). Total cell lysates were immunoblotted with anti-β-tubulin antibody to ensure equal input protein for immunoprecipitation (*lower panel*). One-way ANOVA revealed significant main effect of treatment (F _(3, 8)_ = 146.3; *p* < 0.0001). *B*–*F*, total cell lysates were processed for immunoblot analysis using (*B* and *D*) anti-phospho-Pyk2 ^Y402^ (pPyk2) and anti-Pyk2 antibodies or (*C*, *E*, and *F*) anti-phospho-Src ^Y416^ (pSrc) and anti-Src antibodies. *B*, *C*, and *F*, one-way ANOVA revealed significant main effect of treatment in the presence of (*B*) Pyk2 inhibitor: F _(2, 6)_ = 50.27; *p* = 0.0002, (*C*) SFK inhibitor: F _(2, 6)_ = 59.18; *p* = 0.0001, and (*F*) Pyk2 inhibitor: F _(2, 6)_ = 34.94; *p* = 0.0005. *G* and *H*, rat neuron cultures were treated with 50 μM L-Hcy (4 h) in the presence and absence of GluN2A-NMDAR inhibitor NVP-AAM077 (30 nM) or GluN2B-NMDAR inhibitor Ro25-6981 (5 μM). Total cell lysates were processed for immunoblot analysis using (*G*) anti-pPyk2 and anti-Pyk2 antibodies or (*H*) anti-pSrc and anti-Src antibodies. *I*–*L*, rat neuron cultures were treated with either (*I* and *J*) 50 μM L-Glutamate (0 min, 5 min, and 30 min) or (*K* and *L*) 50 μM L-Hcy (0 min, 5 min, 0.5 h, 1 h, 2 h, and 4 h). Total cell lysates were processed for immunoblot analysis using (*I* and *K*) anti-STEP and anti-tubulin antibodies or (*J* and *L*) anti-pSrc and anti-Src antibodies. *A*, *B*, *C*, and *E*, post hoc analysis by Bonferroni’s multiple-comparisons test shows ∗*p* < 0.01, ∗∗*p* < 0.001, and ∗∗∗*p* < 0.001 between the treatment groups. Values are mean ± SD. Data points represent individual biological replicates. NMDAR, N-methyl-D-aspartate subtype of glutamate receptor; SFK, Src family kinase.

Considerable evidence from earlier studies suggest that the activity of GluN2A-NMDAR is dependent on its phosphorylation at the C-terminal Tyr^1325^ residue by SFKs (30, 31, 32, 33, 34). The activation of SFKs in turn is dependent on its phosphorylation at Tyr^416^, which is determined by activation of Pyk2 through Ca^2+^-dependent autophosphorylation of Pyk2 at Tyr^402^ (35, 36). As such, we next evaluated whether homocysteine-mediated GluN2A-NMDAR activation involves phosphorylation of GluN2A subunit at Tyr^1325^ through activation of Pyk2 and SFKs. For these experiments, in initial studies, neurons were treated with L-homocysteine (50 μM, 4 h) in the presence of Pyk2 inhibitor or SFK inhibitor, followed by immunoprecipitation of GluN2A-NMDAR from cell lysates with anti-GluN2A antibody. Immunoblot analysis of the immune complex with anti-phospho-GluN2A^Y1325^ antibody show significant increase in the phosphorylation of Tyr^1325^ when treated with homocysteine alone (Fig. 2*A*), whereas pharmacological inhibition of either Pyk2 or SFKs attenuates the phosphorylation of Tyr^1325^ (Fig. 2*A*). To determine whether homocysteine treatment could also lead to an increase in phosphorylation of Pyk2 and SFKs, in subsequent studies neuronal cultures were treated with L-homocysteine (50 μM, 4 h) in the presence of either Pyk2 or SFK inhibitor. The extent of phosphorylation of Pyk2 at Tyr^402^ and SFK at Tyr^416^ in cell lysates was evaluated by immunoblotting with anti-pPyk2 or anti-pSrc kinase antibodies, respectively (Fig. 2, *B* and *C*). The results show that treatment with homocysteine alone leads to significant increase in the phosphorylation of both Pyk2 and SFKs, and inhibition of Pyk2 or SFKs blocks homocysteine-mediated increase in their respective phosphorylation (Fig. 2, *B* and *C*). However, inhibition of Pyk2 or SFKs in untreated neurons (controls) has no effect on the phosphorylation of Pyk2 or Src, respectively (Fig. 2, *D* and *E*). Since phosphorylation of SFKs at Tyr^416^ has been reported to be downstream of and dependent on the activation of Pyk2 (35, 36), we further evaluated whether inhibition of Pyk2 phosphorylation can block Src phosphorylation. For this experiment, neurons were treated with L-homocysteine (50 μM, 4 h) in the presence of the Pyk2 inhibitor. Immunoblot analysis with anti-pSrc antibody show that pharmacological inhibition of Pyk2 blocks homocysteine-induced phosphorylation of SFKs at Tyr^416^ (Fig. 2*F*, upper panel). Taken together, these findings suggest that homocysteine-GluN2A-NMDAR–mediated initial Ca^2+^ influx triggers activation of Pyk2, which in turn activates SFKs to help to maintain a feedforward cycle for prolonged GluN2A-NMDAR activation through phosphorylation of Tyr^1325^, resulting in sustained Ca^2+^ influx. To further confirm the role of GluN2A-NMDAR in the phosphorylation of Pyk2 and Src in additional studies, neuronal cultures were treated with L-homocysteine (50 μM, 4 h) in the presence of GluN2A-NMDAR inhibitor NVP-AAM077 (30 nM) or GluN2B-NMDAR inhibitor Ro25-6981 (5 μM). Immunoblot analysis show that inhibition of GluN2A-NMDAR blocks phosphorylation of both Pyk2 and Src, whereas inhibition of GluN2B-NMDARs fails to block phosphorylation of either Pyk2 or Src (Fig. 2, *G* and *H*).

It is further evident from earlier studies that the activity of Pyk2 and SFKs is regulated by the brain-enriched and neuron-specific tyrosine phosphatase STEP (37, 38, 39, 40). STEP_61_, the membrane bound isoform of the STEP-family is ubiquitously expressed in the brain and is the predominant isoform expressed in cultured neurons (41, 42). STEP_61_ is a component of the NMDAR signaling pathway and its function is regulated through phosphorylation of a critical serine residue in the kinase-interacting motif (43, 44). STEP_61_ is basally phosphorylated at this residue, which renders it inactive in terms of its ability to bind to its substrates and dephosphorylate them (43, 44). Dephosphorylation of this serine residue after glutamate-mediated GluN2B-NMDAR stimulation renders STEP_61_ active (43), and active STEP_61_ (dephosphorylated) is typically detected by a downward shift in the mobility of the STEP_61_ protein band (43, 45, 46, 47). To evaluate the phosphorylation status of STEP_61_ after glutamate treatment, neuronal cultures were treated with glutamate (50 μM) for the specified time periods (5 and 30 min), and cell lysates were analyzed by immunoblotting with anti-STEP antibody. As previously reported, in the absence of any stimulation (control), STEP_61_ was found to be primarily phosphorylated (top band), (Fig. 2*I*, upper panel, lane 1), whereas treatment with glutamate decreases the intensity of the top band with a concomitant increase in the intensity of the lower band (Fig. 2*I*, upper panel, lane 3) within 30 min, confirming the dephosphorylation of STEP_61_. To evaluate the phosphorylation status of STEP_61_ following treatment with homocysteine, neuronal cultures were treated with L-homocysteine (50 μM) for specified time periods (5 min, 30 min, 1 h, 2 h, and 4 h). The findings show that the phosphorylated STEP_61_ (upper band) is the predominant isoform present under these conditions, which confirms that homocysteine treatment fails to dephosphorylate STEP_61_ (Figure 2*K*, upper panel). This finding suggests that the lack of activation of STEP_61_ following homocysteine-GluN2A NMDAR stimulation leads to persistent phosphorylation of Pyk2 and SFKs, which in turn helps to maintain prolonged GluN2A-NMDAR phosphorylation and channel activity. To further validate this hypothesis, in additional experiments, we evaluated the time course of SFK phosphorylation after treatment with glutamate (50 μM, 30 min) or L-homocysteine (50 μM, 4 h). The findings show that treatment with glutamate leads to a transient increase in SFK phosphorylation within 5 min of stimulation, whereas treatment with homocysteine leads to a sustained increase in SFK phosphorylation observed throughout the time period of the study (Fig. 2, *J* and *L*). Consistent with this finding, in earlier studies, we also observed that phosphorylation of ERK MAPK, another substrate of STEP, is also distinctly different after treatment of neurons with glutamate and homocysteine (21, 23). While treatment with glutamate (50 μM, 1 h) led to a rapid but transient increase in ERK MAPK phosphorylation within 5 min and returned to basal level by 30 min of stimulation, homocysteine-mediated ERK MAPK phosphorylation remained sustained for the entire period of the study.

### Homocysteine-GluN2A-NMDAR–mediated sustained ERK MAPK phosphorylation is dependent on Pyk2 and SFK activation

**Figure 3:**
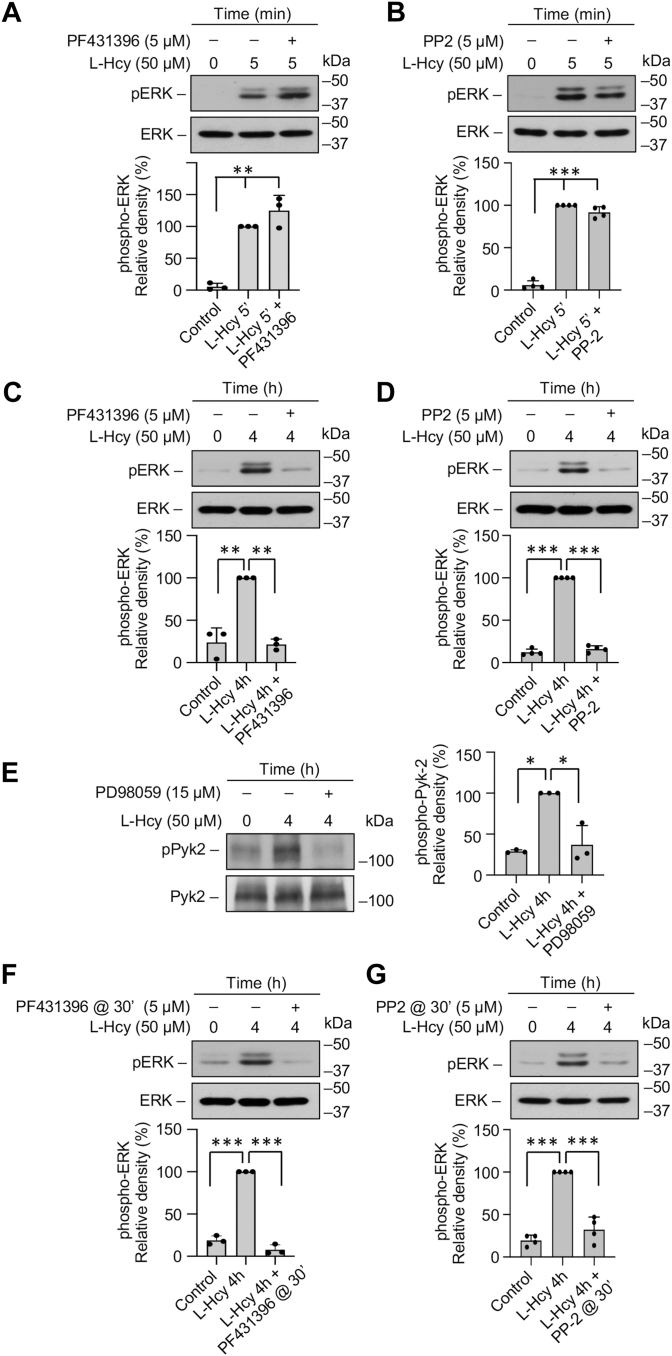
**Homocysteine-induced delayed phosphorylation of ERK MAPK is dependent on Pyk2 and SFK activation.***A*–*G*, rat neuron cultures were treated with 50 μM of L-homocysteine (L-Hcy) for (*A* and *B*) 5 min or (*C*–*G*) 4 h. L-Hcy treatment was carried out in the presence or absence of (*A* and *C*) Pyk2 inhibitor PF431396 (5 μM); (*B* and *D*) SFK inhibitor PP2 (5 μM); or (*E*) ERK MAPK inhibitor PD98059 (10 μM). In some experiments (*F*), Pyk2 inhibitor PF431396 or (*G*) SFK inhibitor PP2 was added 30 min after the onset of L-Hcy treatment. Immunoblot analysis was performed using (*A*, *B*, *C*, *D*, *F*, and *G*) anti-p-ERK and p-ERK antibodies or (*E*) anti-pPyk2 and anti-Pyk2 antibodies. One-way ANOVA revealed significant main effect of treatment in the presence of (*A*) Pyk2 inhibitor: F _(2, 6)_ = 58.46; *p* = 0.0001 (*B*) SFK inhibitor: F _(2, 9)_ = 471.2; *p* < 0.0001, (*C*) Pyk2 inhibitor: F _(2, 6)_ = 54.12; *p* = 0.0001, (*D*) SFK inhibitor: F _(2, 9)_ = 1038; *p* < 0.0001, (*E*) ERK MAPK inhibitor: F _(2, 6)_ = 24.69; *p* = 0.0013, (*F*) Pyk2 inhibitor: F _(2, 6)_ = 376.0; *p* < 0.0001, and (*G*) SFK inhibitor: F _(2, 9)_ = 86.79; *p* < 0.0001. Post hoc analysis by Bonferroni’s multiple-comparisons test shows ∗*p* < 0.01, ∗∗*p* < 0.001, and ∗∗∗*p* < 0.0001 between the treatment groups. Values are mean ± SD. Data points represent individual biological replicates. SFK, Src family kinase.

The sustained increase in ERK MAPK phosphorylation following homocysteine-GluN2A-NMDAR–mediated Ca^2+^ influx observed in earlier studies is characterized by a rapid initial increase (2.5–5 min), followed by a delayed larger increase (4 h), and plays a role in homocysteine-mediated neuronal cell death (20, 21, 23). To evaluate the involvement of Pyk2 and SFKs in mediating the biphasic phosphorylation of ERK MAPK, neuronal cultures were treated with L-homocysteine (50 μM) for 5 min or 4 h in the presence of Pyk2 inhibitor or SFK inhibitor. Immunoblot analysis with anti-pERK antibody shows that treatment with homocysteine alone leads to increase in ERK MAPK phosphorylation at both 5 min and 4 h, which is consistent with the previous findings (20, 21, 23, 24). Pharmacological inhibition of Pyk2 or SFKs has no effect on the initial increase in ERK MAPK phosphorylation at 5 min (Fig. 3, *A* and *B*) but significantly reduces the delayed increase in ERK MAPK phosphorylation at 4 h (Fig. 3, *C* and *D*). This later finding raises the possibility that the initial increase in ERK MAPK phosphorylation leads to Pyk2-mediated SFK activation to maintain GluN2A-NMDAR stimulation, which is essential for sustained ERK MAPK phosphorylation. To test this hypothesis, neurons were treated with L-homocysteine (50 μM, 4 h) in the presence of ERK MAPK inhibitor PD98059 (15 μM), and immunoblot analysis was performed with anti-pPyk2 antibody. The findings show that inhibition of ERK MAPK phosphorylation leads to significant reduction in Pyk2 phosphorylation at 4 h (Fig. 3*E*). To further confirm the role of Pyk2 and SFKs in facilitating the delayed phosphorylation of ERK MAPK by 4 h, in subsequent studies, inhibitors of Pyk2 or SFKs was added 30 min after the onset of homocysteine treatment. Immunoblot analysis show (Fig. 3, *F* and *G*) that delayed administration of either the Pyk2 or SFK inhibitor significantly reduces homocysteine-induced ERK MAPK phosphorylation at 4 h. Taken together, the findings indicate the presence of a feedback loop triggered by the initial increase in ERK MAPK phosphorylation, to maintain Pyk2/Src/GluN2A-NMDAR–mediated sustained ERK MAPK phosphorylation following homocysteine treatment.

**Figure 4:**
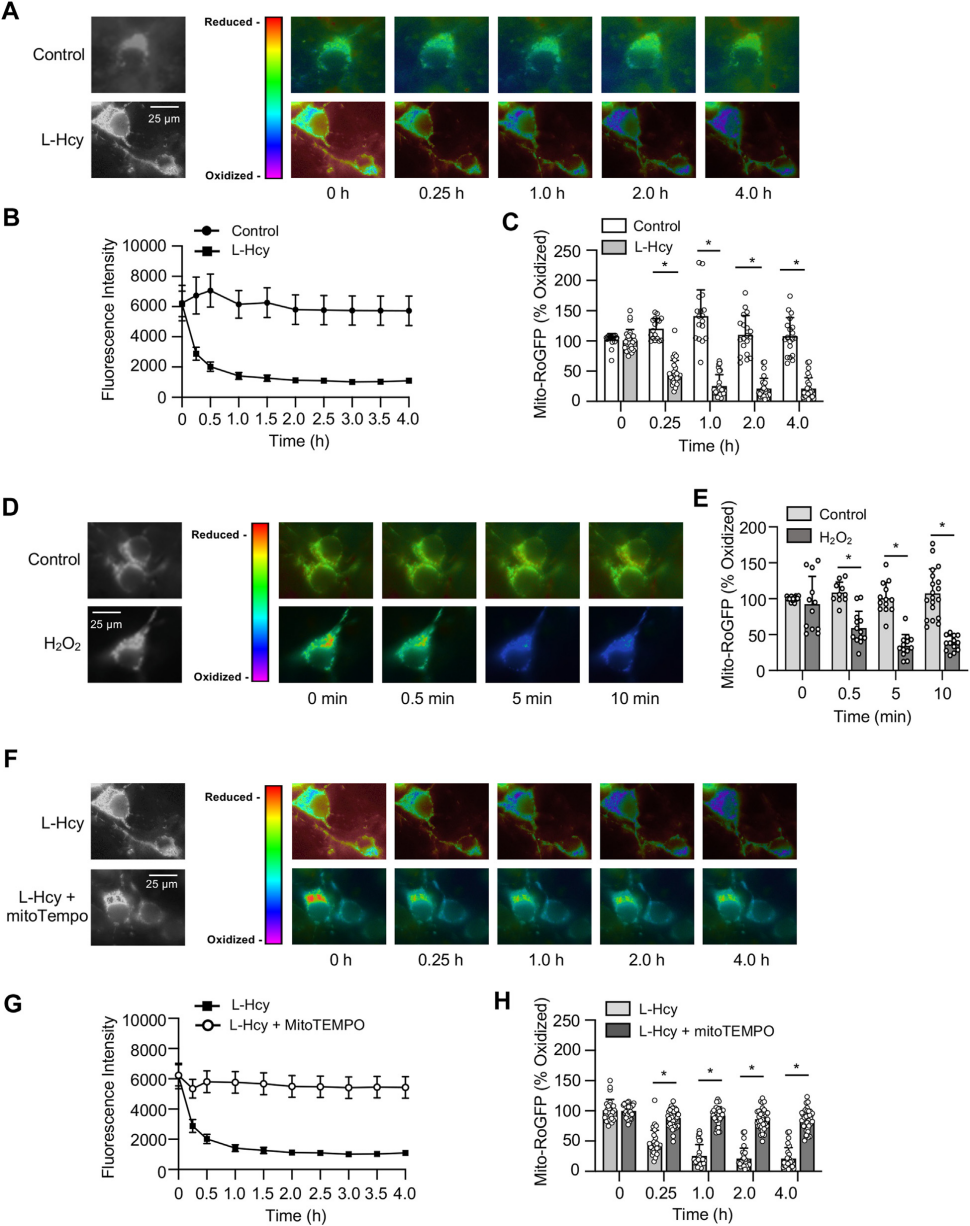
**Homocysteine leads to progressive increase in mitochondrial ROS generation.***A*–*E*, rat neuronal cultures expressing mito-RoGFP were treated with 50 μM of L-homocysteine (L-Hcy, 4 h) or 50 μM H_2_O_2_ (10 min), and loss of fluorescence signal was recorded (excitation 484 nm) at the specified times. *A* and *D*, representative photomicrographs (*black and white*, and *false-color images*) showing temporal loss of fluorescence signal of mito-RoGFP with (*A*) L-Hcy or (*D*) H_2_O_2_ treatment indicating generation of mitochondrial ROS. The representative photomicrographs displaying changes in fluorescence intensity of mito-RoGFP following L-Hcy treatment in Figure 4*A* has also been incorporated in (*F*), and Figure 5, *A* and *D* (as a positive control) for side-by-side visual comparison of the effects of pharmacological inhibitors on L-Hcy–induced changes in fluorescence intensity of mito-RoGFP. *B*, temporal changes in fluorescence intensity (arbitrary units) in control and L-Hcy–treated neurons-expressing mito-RoGFP (mean ± SE). *C* and *E*, quantitative analysis of the percentage decrease in fluorescence intensity of oxidized mito-RoGFP signal in individual cells following L-Hcy or H_2_O_2_ treatment compared to untreated control cells expressed as mean ± SD. Two-way ANOVA followed by Bonferroni’s multiple-comparisons test shows significant effect of treatment, time, and interaction in the presence L-Hcy (treatment effect: F _(1, 44)_ = 192.2; *p* < 0.0001, time effect: F _(1.891, 81.32)_ = 34.38; *p* < 0.0001, interaction: F _(4, 172)_ = 66.21; *p* < 0.0001), and H_2_O_2_ (treatment effect: F _(1, 30)_ = 77.37; *p* < 0.0001, time effect: F _(3, 74)_ = 10.69; *p* < 0.0001, interaction: F _(3, 74)_ = 15.09; *p* < 0.0001). *F*–*H*, neurons-expressing mito-RoGFP were treated with 50 μM of L-Hcy in the presence or absence of mitoTEMPO (5 μM) for 4 h. *F*, representative photomicrographs (*black and white*, and *false-color images*) showing temporal changes in fluorescence intensity of mito-RoGFP. *G*, temporal changes in mito-RoGFP fluorescence intensity (arbitrary units) over time and (*H*) quantitative analysis of percentage increase in reduced mito-RoGFP signal at the specified time points. Two-way ANOVA followed by Bonferroni’s multiple-comparisons test shows significant effect of treatment, time, and interaction (Treatment effect: F _(1, 62)_ = 244.4; *p* < 0.0001, Time effect: F _(1.826, 113.2)_ = 144.7; *p* < 0.0001, Interaction: F _(4, 248)_ = 77.31; *p* < 0.0001). Post hoc analysis shows ∗*p* < 0.0001 between the treatment groups at the given time point. Data points represent individual biological replicates. ROS, reactive oxygen species; SFK, Src family kinase.

**Figure 5:**
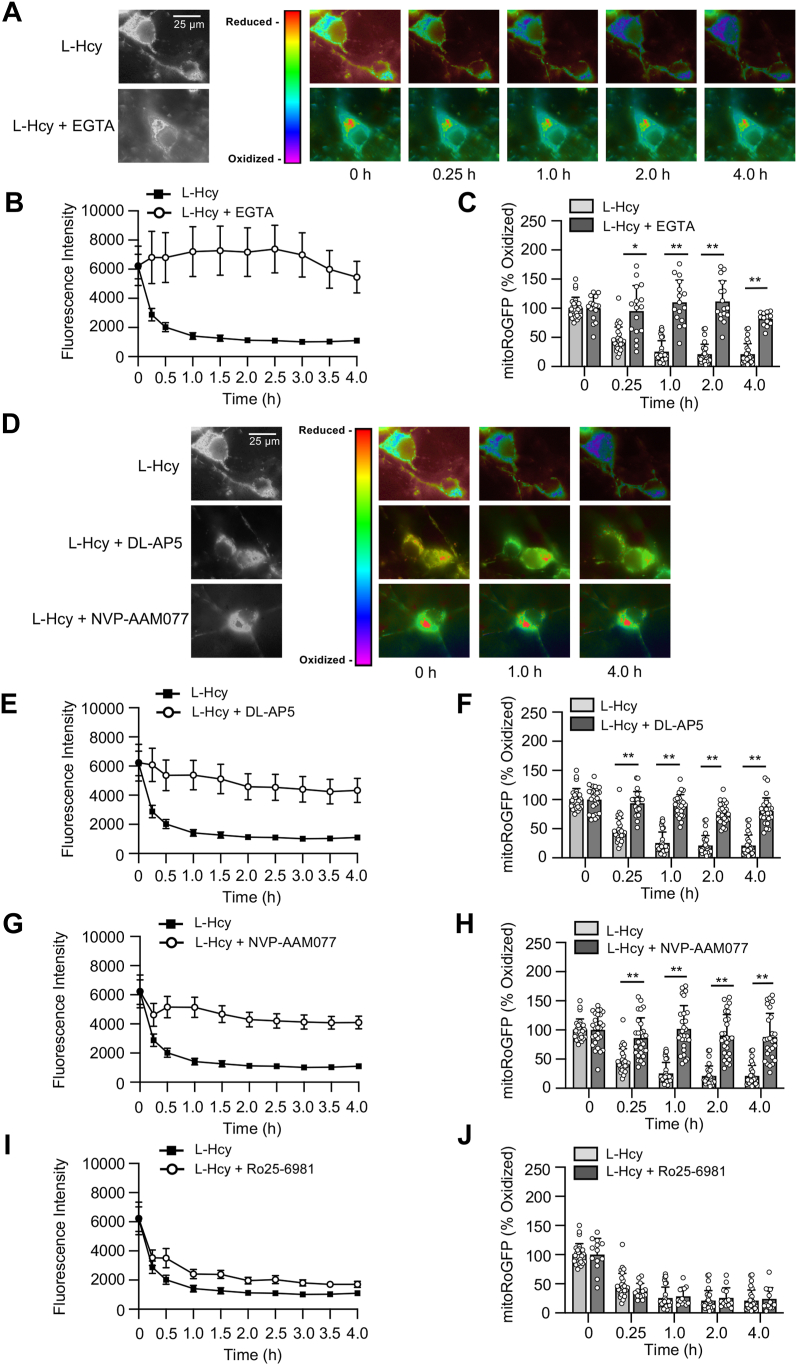
**Homocysteine-induced mitochondrial ROS generation is dependent on GluN2A-NMDAR.***A*–*J*, rat neuronal cultures expressing mito-RoGFP were treated with 50 μM of L-homocysteine (L-Hcy, 4 h) in the presence or absence of (*A*–*C*) EGTA (2.5 μM), (*D*–*F*) DL-AP5 (200 μM), (*D*, *G*, and *H*) NVP-AAM077 (30 nM), or (*I* and *J*) Ro25-6951 (5 μM). *A* and *D*, representative photomicrographs (*black* and *white*, and *false-color images*) showing temporal changes in fluorescence intensity of mito-RoGFP. *B*, *E*, and *G*, temporal changes in mito-RoGFP fluorescence intensity (arbitrary units) over time in the presence of (*B*) EGTA, (*E*) DL-APV, and (*G*) NVP-AAM077 expressed as mean ± SE. *C*, *F*, and *H*, quantitative analysis (two-way ANOVA followed by Bonferroni’s multiple-comparisons test) of the percentage increase in fluorescence intensity of reduced mito-RoGFP signal in the presence of (*C*) EGTA, (*F*) DL-APV, and (*H*) NVP-AAM077 expressed as mean ± SD. Data shows significant effect of treatment, time, and interaction in the presence EGTA (treatment effect: F _(1, 41)_ = 125.8; *p* < 0.0001, time effect: F _(2.026, 81.54)_ = 26.76; *p* < 0.0001, interaction: F _(4, 161)_ = 30.22; *p* < 0.0001), DL-APV (treatment effect: F _(1, 51)_ = 179.7; *p* < 0.0001, time effect: F _(2.033, 102.2)_ = 81.99; *p* < 0.0001, interaction: F _(4, 201)_ = 31.93; *p* < 0.0001) and NVP-AAM077 (treatment effect: F _(1, 52)_ = 87.34; *p* < 0.0001, time effect: F _(1.518, 78.93)_ = 34.10; *p* < 0.0001, interaction: F _(4, 208)_ = 24.11; *p* < 0.0001). *I*, temporal profile of changes in mito-RoGFP fluorescence intensity (arbitrary units) in the presence of Ro25-6951 expressed as mean ± SE and (*J*) quantitative analysis of percentage change in fluorescence intensity of oxidized mito-RoGFP signal at the given time points does not show significant effect of treatment and interaction in the presence Ro25-6981(treatment effect: F _(1, 38)_ = 0.0008; *p* = 0.9775, Time effect: F _(1.604, 60.96)_ = 158.1; *p* < 0.0001, interaction: F _(4, 152)_ = 1.09; *p* = 0.3593). Post hoc analysis shows ∗*p* < 0.01 and ∗∗*p* < 0.0001 between the treatment groups at the given time point. Data points represent individual biological replicates. Mito-RoGFP, mitochondria-targeted redox-sensitive GFP; NMDAR, N-methyl-D-aspartate subtype of glutamate receptor; ROS, reactive oxygen species.

**Figure 6:**
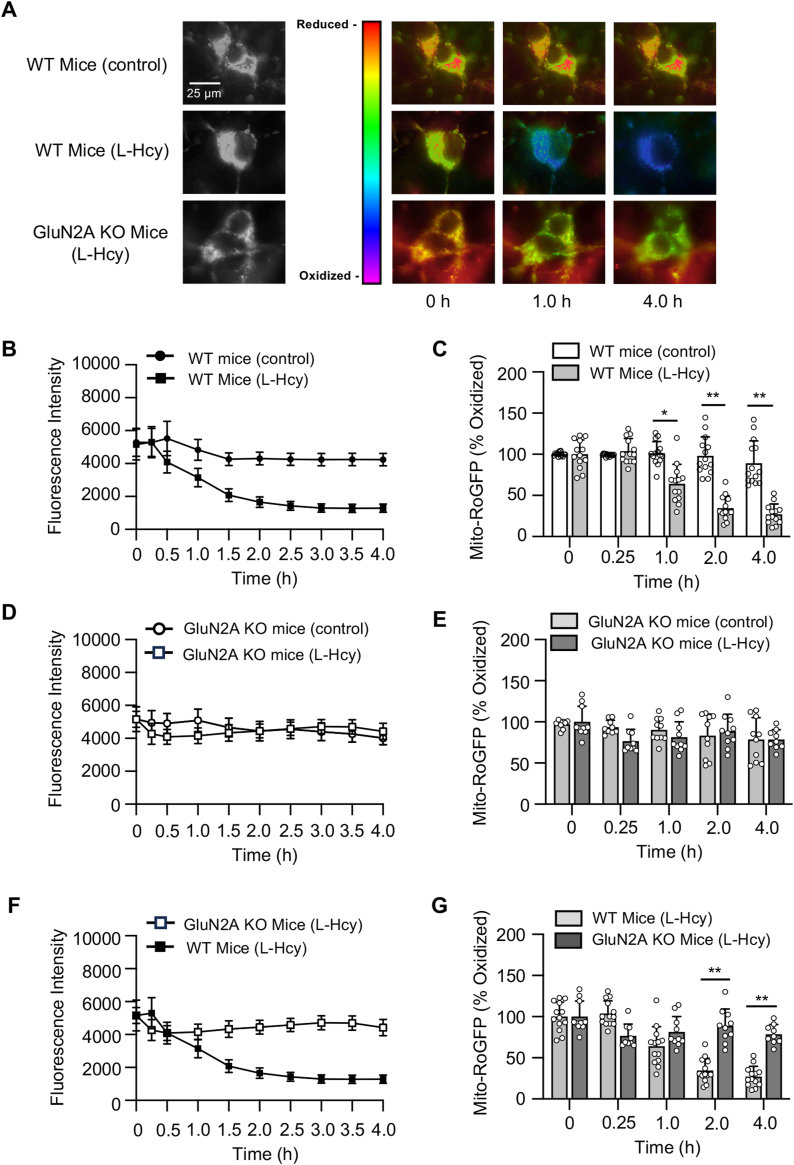
**Knockdown of GluN2A subunit blocks homocysteine-induced mitochondrial ROS generation.***A*–*G*, neuronal cultures from WT and GluN2A KO mice–expressing mito-RoGFP were treated with or without 50 μM of L-homocysteine (L-Hcy, 4 h) and changes in fluorescence signal of mito-RoGFP was recorded at the specified time points. *A*, representative photomicrographs (*black* and *white*, and *false-color images*) showing temporal changes in fluorescence intensity of mito-RoGFP. *B*, *D*, and *F*, temporal profile of changes in mito-RoGFP fluorescence intensity (arbitrary units) over time in (*B*) WT control and L-Hcy–treated WT neurons, (*D*) GluN2A-KO control and L-Hcy–treated GluN2A-KO neurons, and (*F*) L-Hcy–treated WT and L-Hcy–treated GluN2A KO neurons expressed as mean ± SE. *C*, *E*, and *G*, quantitative analysis of the percentage change in fluorescence intensity of oxidized mito-RoGFP signal at the given time points in (*C*) L-Hcy–treated WT neurons compared to vehicle-treated WT neurons (control), (*E*) L-Hcy–treated GluN2A-KO neurons compared to vehicle-treated GluN2A-KO neurons (control), and (*G*) L-Hcy–treated WT neurons compared to L-Hcy–treated GluN2A KO neurons (control) expressed as mean ± SD. Two-way ANOVA followed by Bonferroni’s multiple-comparisons test shows significant effect of treatment, time, and interaction in (*C*) treatment effect: (F _(1, 25)_ = 51.48; *p* < 0.0001), time effect: (F _(2.744, 68.61)_ = 50.05; *p* < 0.0001), interaction: (F _(4, 100)_ = 35.31; *p* < 0.0001) and (*G*) treatment effect: (F _(1, 21)_ = 18.05; *p* = 0.0004), time effect: (F _(2.924, 59.94)_ = 35.68; *p* < 0.0001), interaction: (F _(4, 82)_ = 28.32; *p* < 0.0001). *E*, two-way ANOVA followed by Bonferroni’s multiple-comparisons test does not show significant effect of treatment and interaction (treatment effect: F _(1, 18)_ = 0.498; *p* = 0.4894, time effect: F _(4, 69)_ = 4.946; *p* = 0.0015, interaction: F _(4, 69)_ = 2.412; *p* = 0.0572). Post hoc analysis shows ∗*p* < 0.001 and ∗∗*p* < 0.0001 between the treatment groups at the given time point. Data points represent individual biological replicates. Mito-RoGFP, mitochondria-targeted redox-sensitive GFP; ROS, reactive oxygen species.

**Figure 7:**
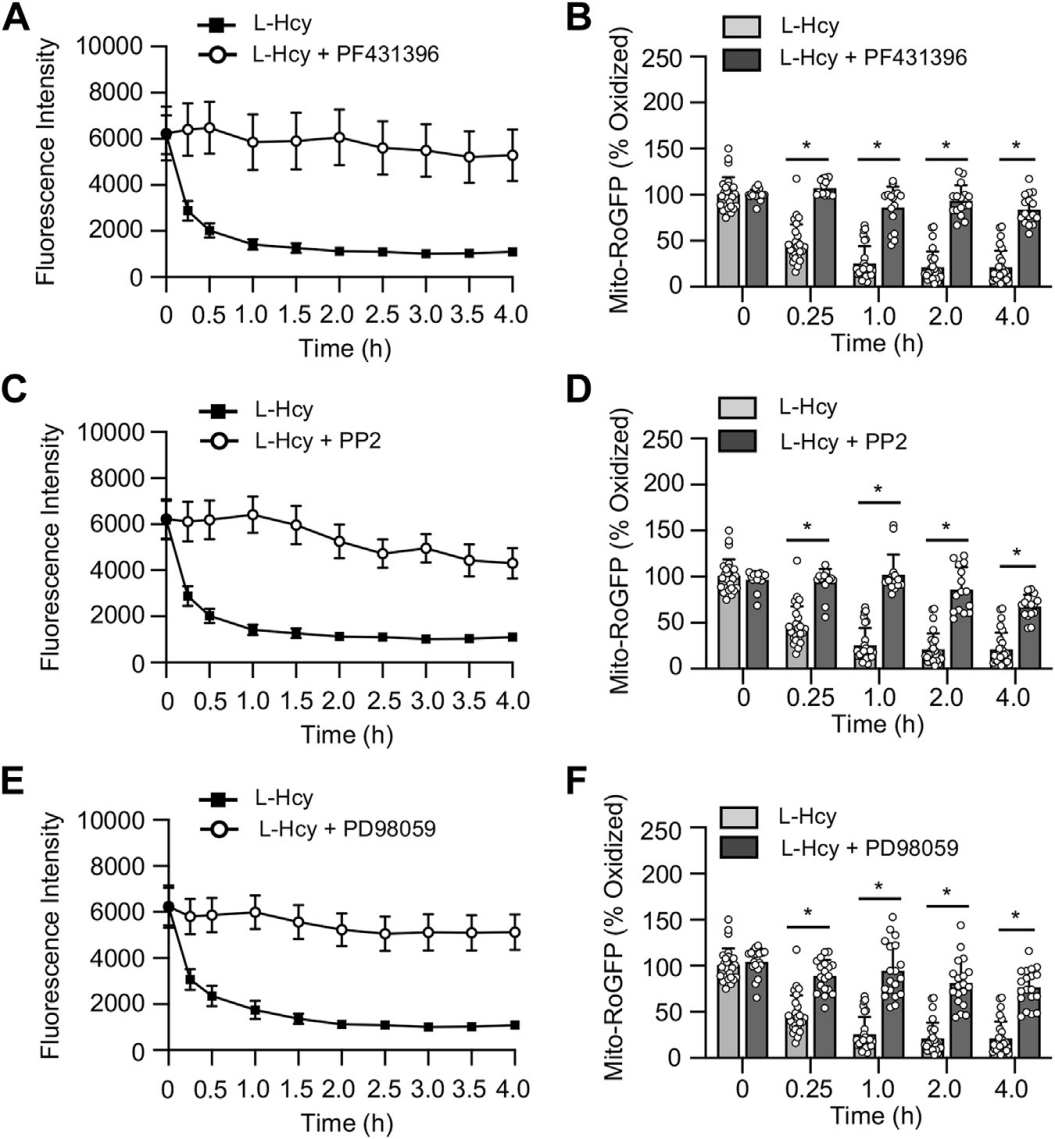
**Homocysteine-induced mitochondrial ROS generation comprises a feedback loop involving activation of ERK MAPK.** Rat neuronal cultures expressing mito-RoGFP were treated with 50 μM of L-homocysteine (L-Hcy, 4 h) in the presence or absence (*A* and *B*) Pyk2 inhibitor PF431396, (*C* and *D*) SFK inhibitor PP2, or (*E* and *F*) ERK MAPK inhibitor PD98059 (15 μM). *A*, *C*, and *E*, temporal profile of changes in mito-RoGFP fluorescence intensity (arbitrary units) over time expressed as mean ± SE. *B*, *D*, and *F*, quantitative analysis (two-way ANOVA followed by Bonferroni’s multiple-comparisons test) of the percentage change in fluorescence intensity of oxidized mito-RoGFP signal at the given time points expressed as mean ± SD. Data shows significant effect of treatment, time, and interaction in the presence of PF431396 (treatment effect: F _(1, 41)_ = 195.8; *p* < 0.0001, time effect: F _(2.487, 102)_ = 74.82; *p* < 0.0001, interaction: F _(4, 164)_ = 39.80; *p* < 0.0001), PP2 (treatment effect: F _(1, 40)_ = 165.9; *p* < 0.0001, time effect: F _(2.730, 109.2)_ = 62.13; *p* < 0.0001, interaction: F _(4, 160)_ = 34.37; *p* < 0.0001) and PD98059 (treatment effect: F _(1, 45)_ = 117.2; *p* < 0.0001, time effect: F _(1.937, 84.74)_ = 81.53; *p* < 0.0001, interaction: F _(4, 175)_ = 29.47; *p* < 0.0001). Post hoc analysis shows ∗*p* < 0.0001 between the treatment groups at the given time point. Data points represent individual biological replicates. Mito-RoGFP, mitochondria-targeted redox-sensitive GFP; ROS, reactive oxygen species; SFK, Src family kinase.

**Figure 8:**
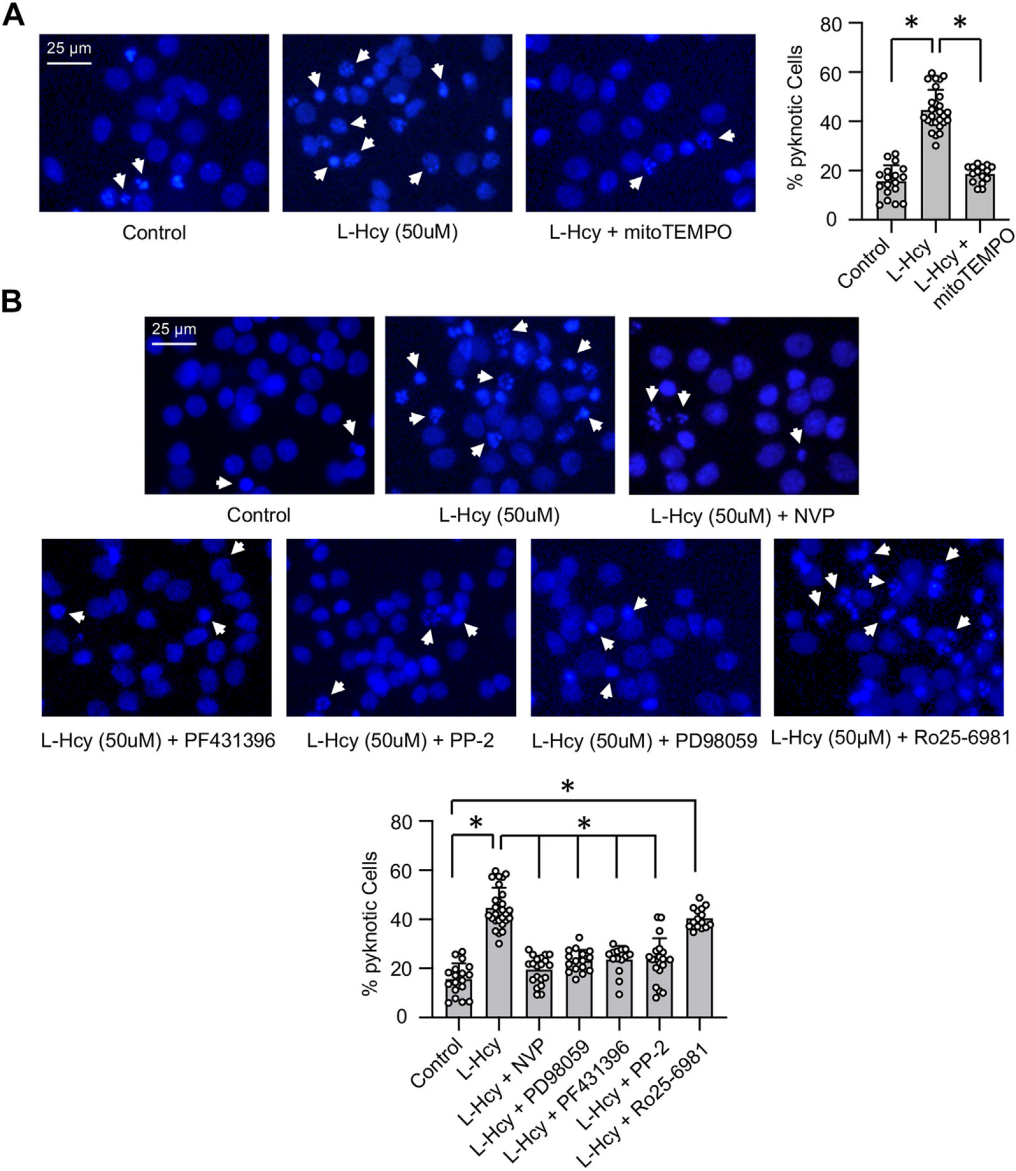
**Homocysteine-GluN2A-NMDAR–induced neurotoxicity involves Pyk2 and SFK activation and mitochondrial ROS generation.***A* and *B*, rat neuronal cultures were treated with 50 μM of L-homocysteine (L-Hcy) for 18 h in the presence of (*A*) mitoTEMPO (5 μM); (*B*) NVP-AAM077 (30 nM), PD98059 (15 μM), PF431396 (5 μM), PP2 (5 μM), or Ro25-6981 (5 μM). Representative photomicrographs of neurons stained with nuclear stain Hoechst 33342 (*blue*) showing pyknotic DNA (indicated with *arrows*). The percentage of neurons with pyknotic nuclei are expressed as mean ± SD. One-way ANOVA reveal significant main effect of treatment on cell death in the presence of (*A*) mitoTEMPO: F _(2, 57)_ = 124.8; *p* < 0.0001 and (*B*) other pharmacological inhibitors: F _(6, 123)_ = 55.95; *p* < 0.0001. Post hoc analysis by Bonferroni’s multiple-comparisons test shows ∗*p* < 0.0001 between the treatment groups. Data points represent individual biological replicates. NMDAR, N-methyl-D-aspartate subtype of glutamate receptor; ROS, reactive oxygen species; SFK, Src family kinase.

**Figure 9:**
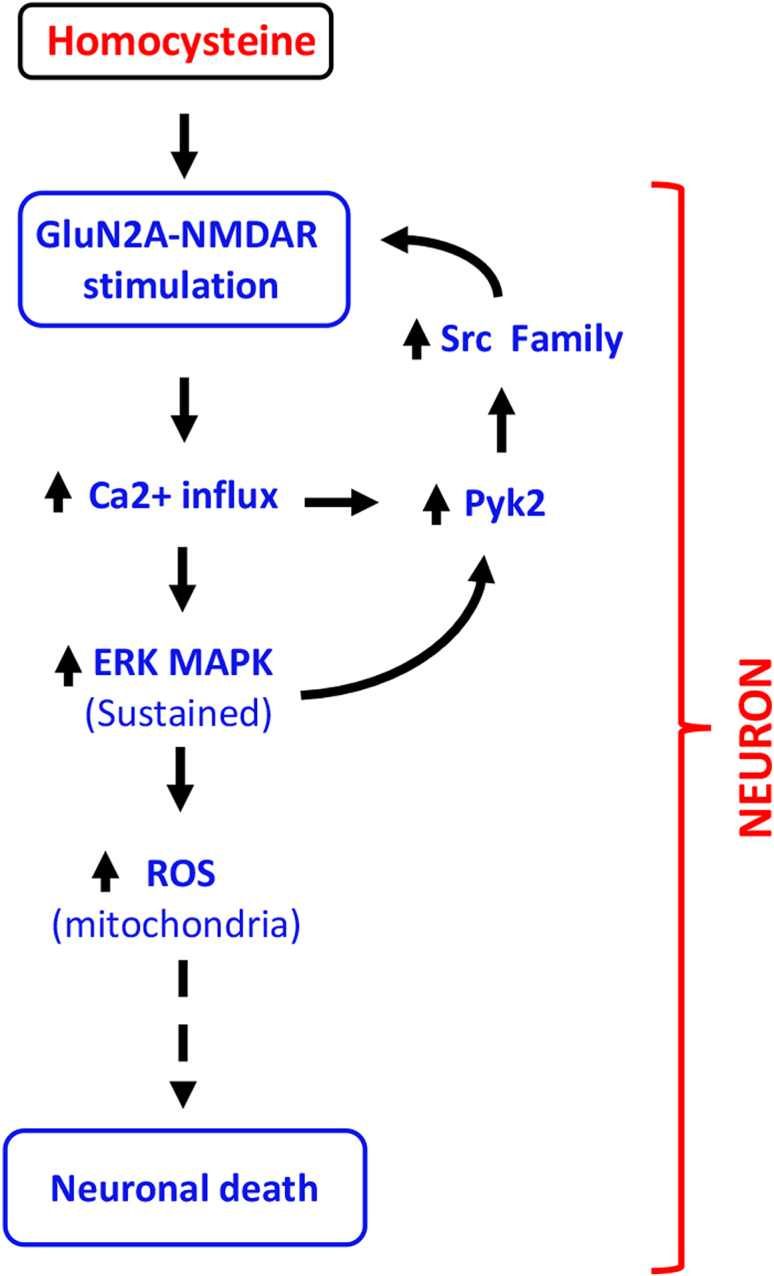
**Schematic representation of homocysteine-GluN2A-NMDAR–mediated intracellular signaling.** Homocysteine-mediated GluN2A-NMDAR stimulation leads to initial Ca^2+^ influx, triggering sequential activation of Pyk2 and Src family kinase. This in turn leads to phosphorylation of GluN2A-NMDAR-Tyr^1325^, thereby enhancing channel activity and further influx of Ca^2+^. The GluN2A-NMDAR–mediated initial influx of Ca^2+^ also leads to sustained activation of ERK MAPK that causes mitochondrial ROS generation. The sustained ERK MAPK activation also promotes Pyk2/Src kinase activation further contributing to the cycle of signaling events that eventually results to neuronal death. NMDAR, N-methyl-D-aspartate subtype of glutamate receptor; ROS, reactive oxygen species; SFK, Src family kinase.

### Homocysteine-GluN2A-NMDAR–mediated ERK MAPK activation leads to sustained mitochondrial ROS generation that contributes to neuronal death

Previous studies have indicated that exposure to elevated levels of homocysteine leads to ROS generation in neuronal cells (13, 48). As mitochondria is a major source of ROS in brain cells, we next monitored long-term changes in oxidative status selectively in the mitochondria by live-cell imaging, utilizing a redox-sensitive fluorescent protein-based biosensor, mito-RoGFP targeted to the mitochondrial matrix (49). For these studies, neuronal cultures were infected with an adenovirus-encoding mito-RoGFP and the infected cells were treated with vehicle (saline) or L-homocysteine (50 μM), followed by assessment of fluorescent intensity of mito-RoGFP by live-cell imaging for 4 h, at specified intervals. The representative photomicrographs presented in Figure 4*A* shows that in vehicle-treated (control) cells, the fluorescence intensity of mito-RoGFP (in false-color maps) remains unchanged over time, whereas in homocysteine treated cells, there is a time-dependent decrease in mito-RoGFP fluorescence intensity, an indicator of increased mitochondrial ROS generation (49). Assessment of mito-RoGFP fluorescence intensity over time presented as a line graph (Fig. 4*B*) and the extent of mito-RoGFP oxidation at selective time points (15 min, 1 h, 2 h, and 4 h) presented in the corresponding bar graphs indicate that homocysteine treatment leads to progressive and significant increase in mitochondrial ROS generation, whereas no change in mitochondrial redox status is observed in neurons treated with vehicle. To ensure that the observed decrease in fluorescence intensity was due to generation of ROS, in subsequent studies, neurons infected with the adenovirus encoding mito-RoGFP were exposed to H_2_O_2_ (50 μM, 10 min), the most abundant form of ROS produced by mitochondria (50). The representative photomicrographs (Fig. 4*D*) and quantitative analysis (Fig. 4*E*) show that fluorescence intensity of mito-RoGFP decreases significantly within minutes of exposure to H_2_O_2_. To further confirm that mitochondria is the primary source of homocysteine-induced ROS generation, mito-RoGFP–expressing neurons were treated with L-homocysteine (50 μM, 4 h) in the presence or absence of MitoTEMPO (5 μM), a mitochondria-targeted antioxidant. The representative photomicrographs (Fig. 4*F*) and the quantitative analysis (Fig. 4, *G* and *H*) show that treatment with MitoTEMPO blocks homocysteine-induced reduction in fluorescence intensity of mito-RoGFP.

Next, we sought to evaluate the intracellular signaling pathway that is involved in homocysteine-induced mitochondrial ROS generation. To determine whether Ca^2+^ influx is necessary for ROS generation, mito-RoGFP–expressing neurons were treated with L-homocysteine (50 μM) and maintained in a calcium-free media containing 0.5 mM EGTA to chelate any residual extracellular calcium. The representative photomicrographs (Fig. 5*A*) and quantitative analysis of mito-RoGFP fluorescence intensity (Fig. 5, *B* and *C*) show that, in the absence of extracellular Ca^2+^, the decrease in homocysteine-induced fluorescence intensity of mito-RoGFP is completely blocked. To evaluate the role of GluN2A-NMDARs in homocysteine-induced mitochondrial ROS generation, mito-RoGFP–expressing neurons were treated with L-homocysteine (50 μM, 4 h) in the presence of NMDAR inhibitor or GluN2A-NMDAR inhibitor. The representative photomicrographs (Fig. 5*D*) and quantitative analysis of mito-RoGFP fluorescence intensity show that both DL-APV (Fig. 5, *E* and *F*) and NVP-AAM077 (Fig. 5, *G* and *H*) block homocysteine-induced decrease in mito-RoGFP fluorescence intensity. In subsequent studies, mito-RoGFP–expressing neurons were treated with L-homocysteine (50 μM, 4 h) in the presence of the GluN2B-NMDAR inhibitor. The analysis of mito-RoGFP fluorescent intensity show that GluN2B-NMDAR inhibition fails to block homocysteine-induced decrease in mito-RoGFP fluorescence intensity (Fig. 5, *I* and *J*), eliminating the role of GluN2B-NMDARs in homocysteine-induced mitochondrial ROS generation. To further confirm the specific role of GluN2A-NMDARs in homocysteine-induced mitochondrial ROS generation, in subsequent studies, mito-RoGFP was expressed in cortical neuronal cultures obtained from WT and GluN2A-KO mice. The cells were then treated with vehicle (saline) or L-homocysteine (50 μM, 4 h), followed by live-cell imaging to monitor changes in mito-RoGFP fluorescence intensity. The representative photomicrographs (Fig. 6*A*) and quantitative analysis (Fig. 6, *B* and *C*) show that the treatment of neuronal cultures obtained from WT mice with homocysteine leads to a significant decrease in mito-RoGFP fluorescence intensity over time, as compared to vehicle-treated WT control, which is consistent with our findings in neuronal cultures obtained from rat brain (Fig. 4*A*). However, neurons obtained form GluN2A-KO mice did not show significant decrease in mito-RoGFP fluorescence intensity, following homocysteine treatment (Fig. 6, *D* and *E*). Comparison of mito-RoGFP fluorescence intensity in neurons obtained from WT and GluN2A-KO mice treated with homocysteine shows significant decrease in fluorescence intensity in WT neurons over time (Fig. 2, *F* and *G*). Together, these findings confirm the role of GluN2A-NMDAR in homocysteine-induced mitochondrial ROS generation in neurons.

To delineate the role of Pyk2 and SFKs in homocysteine-induced mitochondrial ROS generation, in subsequent studies, mito-RoGFP–expressing neurons obtained from rats were treated with L-homocysteine (50 μM) in the presence or absence of the Pyk2 inhibitor or SFK inhibitor. Quantitative analysis of mito-RoGFP fluorescence intensity shows that the inhibition of either Pyk2 or SFKs blocks homocysteine-induce decrease in mito-RoGFP fluorescence intensity (Fig. 7, *A*–*D*). To further evaluate the role of ERK MAPK in homocysteine-induced mitochondrial ROS generation, mito-RoGFP–expressing neurons were treated with L-homocysteine (50 μM) in the presence or absence of the ERK MAPK inhibitor. The findings show that treatment with ERK MAPK inhibitor also blocks homocysteine-induce decrease in mito-RoGFP fluorescence intensity (Fig. 7, *E* and *F*). Collectively, these findings demonstrate the role of GluN2A-NMDAR–mediated Pyk2, SFK, and ERK MAPK activation in homocysteine-induced mitochondrial ROS generation.

To evaluate the role of mitochondrial ROS generation in homocysteine-mediated neurotoxicity, neuronal cultures were treated with L-homocysteine (50 μM, 18 h) in the presence of mitoTEMPO, and cell death was assessed by Hoechst DNA staining. The representative photomicrographs and quantitative analysis of the pyknotic nuclei show significant reduction in homocysteine-induced neuronal cell death in the presence of mitoTEMPO (Fig. 8*A*). Since GluN2A-NMDAR–mediated activation of Pyk2/SFK/ERK MAPK signaling cascade contributes to homocysteine-induced mitochondrial ROS generation, we further evaluated their role in homocysteine-dependent neurotoxicity. For these studies, neuronal cultures were treated with L-homocysteine (50 μM) in the presence of GluN2A-NMDAR inhibitor, Pyk2 inhibitor, SFK inhibitor or ERK MAPK inhibitor followed by cell death assay. Figure 8*B* shows that the inhibition of both GluN2A-NMDAR and ERK MAPK leads to significant reduction in neuronal cell death, which is consistent with our earlier findings (21, 23). In addition, pharmacological inhibition of Pyk2 and SFK attenuates homocysteine-mediated neuronal cell death. In additional studies, neuronal cultures were treated with L-homocysteine (50 μM) in the presence of Ro25-6981 to evaluate the role of GluN2B-NMDAR in homocysteine-dependent neurotoxicity. The findings show that the inhibition of GluN2B-NMDARs fails to reduce homocysteine-induced neuronal cell death (Fig. 8*B*).

## Discussion

The findings from the current study demonstrate for the first time the role of GluN2A-NMDARs in mitochondrial ROS generation and provide a critical insight into the molecular mechanisms underlying homocysteine-induced mitochondrial ROS generation and neurotoxicity (Fig. 9). We identified a unique signaling cascade where homocysteine-GluN2A-NMDAR–mediated initial Ca^2+^ influx and rapid activation of ERK MAPK leads to sequential phosphorylation and activation of Pyk2 kinase and SFKs. In contrast to the NMDAR agonist glutamate, homocysteine fails to activate the regulatory phosphatase STEP_61_, which is known to limit the duration of Pyk2 and SFKs activation upon NMDAR stimulation, thus enabling the activation of a positive feedback loop where unregulated Pyk2 and SFKs activation helps to prolong GluN2A-NMDAR stimulation and continued Ca^2+^ influx, resulting in sustained ERK MAPK phosphorylation (Fig. 9). Such sustained ERK MAPK phosphorylation is essential for homocysteine-GluN2A-NMDAR–mediated increased mitochondrial ROS generation. The study highlights the involvement of a complex signaling cascade in GluN2A-NMDAR–mediated mitochondrial ROS generation that results in neuronal death (Fig. 9).

Reversible phosphorylation of NMDAR subunits modulates channel function and its downstream signaling pathways (31). Phosphorylation of specific NMDAR subunits are known to upregulate, while dephosphorylation of these subunits downregulate NMDAR channel function. Specifically, the SFKs have been shown to play a critical role in upregulating glutamate-mediated NMDAR channel activity (31). Electrophysiological studies have showed that intracellular application of phosphorylated Src kinase during patch clamp recording enhanced NMDAR currents, while application of protein tyrosine kinase inhibitors depressed NMDAR currents and reduced intracellular Ca^2+^ concentrations (33, 51, 52, 53). Similarly, bath application of protein tyrosine phosphatase inhibitors enhanced NMDAR currents, while introducing exogenous protein tyrosine phosphatase suppressed NMDAR currents (54). Collectively, these findings indicate that any disbalance between the activity of the tyrosine kinases and tyrosine phosphatases could lead to dysregulation of NMDAR channel function, thereby triggering intracellular signaling cascades that are detrimental to neuronal survival. Biochemical studies have further shown that SFKs phosphorylate several tyrosine residues in the cytoplasmic tail of GluN2A-NMDAR (Tyr^842^, Tyr^1292^, Tyr^1325^, and Tyr^1387^) and GluN2B-NMDAR (Tyr^1252^, Tyr^1336^, and Tyr^1472^), resulting in receptor stabilization on the cell surface, channel activation, and influx of Ca^2+^ (31, 55). In this context, earlier studies have shown that while Fyn, a member of the SFKs can phosphorylate both GluN2A and GluN2B-NMDAR in the postsynaptic densities of rat forebrain, it selectively modulates the activity of GluN2B NMDAR (40, 55, 56, 57, 58, 59, 60, 61, 62, 63). On the other hand, Src, another member of the SFKs, phosphorylates multiple tyrosine residues in GluN2A-NMDAR to activate the receptor complex (64, 65, 66). In addition to SFKs, Pyk2 is also involved in upregulating NMDAR surface expression, phosphorylation of Tyr^1472^ in GluN2B-NMDAR, association of GluN2B-NMDAR subunits with postsynaptic density-95 (PSD-95) scaffolding protein, and enhancing NMDAR currents (35, 67, 68, 69, 70, 71). Evidence further suggest that Pyk2-mediated NMDAR activation requires Src kinase activation. Mutational studies along with electrophysiological analysis have shown that sequential activation of Pyk2 and SFKs leads to tyrosine phosphorylation of NMDARs and enhance its function (35). In the absence of a stimulus, Pyk2 remains in a closed conformation due to intramolecular interactions between different domains of Pyk2. Following glutamate-NMDAR stimulation, influx of Ca^2+^ leads to dissociation of the intramolecular interactions in Pyk2, allowing it to bind to Ca^2+^/calmodulin and facilitating its clustering to PSD-95. This results in autophosphorylation of Pyk2 at Tyr^402^, which in turn promotes high-affinity binding of the activated Pyk2 to the SH2 domain of SFKs. Such binding disrupts the intramolecular interactions in SFKs that helps maintain SFKs in a low-activity state, thereby allowing phosphorylation of SFKs at Tyr^416^. The activated SFKs subsequently phosphorylate NMDAR subunits to enhance NMDAR channel activity (72, 73, 74, 75). Although multiple evidence suggest that glutamate-mediated NMDAR stimulation leads to the formation of functional NMDAR complex between Pyk2/Src/PSD95 and either GluN2A- or GluN2B-NMDARs, most of the intracellular signaling cascades activated following binding of Pyk2/Src/PSD95 to NMDARs described till date involves GluN2B-NMDAR stimulation. Furthermore, several reports now demonstrate that although homocysteine is an agonist of NMDAR, the effect of homocysteine-mediated NMDAR stimulation is quite different from glutamate-mediated NMDAR stimulation (20, 21, 23). While glutamate mediates its effects by stimulating both GluN2A and GluN2B NMDARs, homocysteine mediates its effects by stimulating primarily the GluN2A-NMDARs. More importantly, in contrast to glutamate, homocysteine-induced neuronal death is mediated through GluN2A-NMDAR stimulation (20, 21, 23). In this context, the current study delineates the intracellular signaling mechanism involved in homocysteine-mediated sustained GluN2A-NMDAR stimulation. The findings show that homocysteine-mediated initial influx of Ca^2+^ through GluN2A-NMDARs triggers autophosphorylation and activation of Pyk2. Active Pyk2 facilitates increased phosphorylation of SFKs, which in turn leads to phosphorylation of GluN2A-NMDAR at Tyr^1325^, thereby increasing channel activity and further influx of Ca^2+^. The continuity of this cycle of events leads to sustained influx of Ca^2+^ through GluN2A-NMDAR and could be attributed to the lack of activation of an inhibitory tyrosine phosphatase that could counter Pyk2 and/or SFKs to decrease tyrosine phosphorylation of GluN2A-NMDARs.

Earlier studies indicate that several protein tyrosine phosphatases, including PTPα, PTPMEG, PTP1D, and STEP_61_, are components of the NMDAR complex and play a role in regulating NMDAR phosphorylation status and activity (39, 60, 76, 77, 78). Although the association of PTPα, PTPMEG, and PTP1D with the NMDAR complex has been correlated with tyrosine phosphorylation and upregulation of NMDAR activity (31), STEP_61_ is known to downregulate NMDAR function by opposing the activation of Pyk2 and SFKs. After the glutamate-mediated NMDAR stimulation, the activation of STEP_61_ in neurons leads to dephosphorylation of Tyr^402^ in Pyk2 (38), which in turn downregulates the activation of SFKs and NMDAR function. Consistent with this finding, genetic deletion of STEP in neurons has been shown to increase phosphorylation of Try^402^ in Pyk2 (79). In addition, active STEP has also been shown to inhibit glutamate-NMDAR–mediated activation of SFKs through dephosphorylation of Tyr^420^ (39, 40). However, our findings now show that homocysteine-mediated NMDAR activation fails to activate STEP_61_, which is not surprising as homocysteine preferentially stimulates the GluN2A-NMDAR (20, 22, 23), whereas activation of STEP has been shown to be downstream of glutamate-mediated GluN2B-NMDAR stimulation (9, 45). Therefore, in the absence of an active tyrosine phosphatase to limit the phosphorylation of Pyk2 and SFKs, exposure to homocysteine prolongs GluN2A-NMDAR channel activity resulting in sustained Ca^2+^ influx.

In addition to Pyk2 and SFKs, ERK MAPK is also a known substrate of STEP. It has been shown that the duration of ERK MAPK activation after glutamate-NMDAR stimulation is regulated through dephosphorylation of Tyr^204^ in ERK MAPK by active STEP, resulting in transient ERK MAPK activation (43). The magnitude and duration of ERK MAPK phosphorylation determines whether neurons survive or die (80). Glutamate-NMDAR–mediated transient ERK MAPK activation has been implicated in neuronal survival and synaptic plasticity (2, 43, 81, 82), whereas sustained ERK MAPK activation following exposure to a diverse array of extracellular stimuli has been shown to cause neuronal death (83, 84, 85). However, the precise role of NMDAR subunits in such sustained ERK MAPK activation is still not well understood. In this context, our earlier studies have demonstrated that homocysteine mediated GluN2A-NMDAR stimulation leads to sustained ERK MAPK phosphorylation that results in neuronal death (20, 21). The current study expands on these earlier findings to delineate the intracellular signaling pathway involved in such sustained ERK MAPK activation. The findings show that the activation of the upstream kinases Pyk2 and SFKs facilitates GluN2A-NMDAR–mediated Ca^2+^ influx, which in conjunction with the lack of activation of STEP_61_ contributes to the sustained ERK MAPK phosphorylation observed in our study.

Another potentially important finding from our study is the role of GluN2A-NMDARs in mitochondrial ROS generation. Earlier studies have implicated the role of ROS in homocysteine-induced neuronal death (13, 48, 86, 87). However, the precise mechanism and the cellular source of such ROS generation are still unclear. Using a redox-sensitive genetic probe targeted to the mitochondrial matrix (mito-RoGFP) and mitochondria-targeted superoxide scavenger (mitoTempo), our findings now demonstrate that neuronal exposure to elevated levels of homocysteine leads to mitochondrial ROS generation. The findings also demonstrate a crucial role of GluN2A-NMDAR in homocysteine-mediated mitochondrial ROS generation. Although the current study highlights the role of GluN2A-NMDARs in homocysteine-induced mitochondrial ROS generation, an earlier study has shown that inhibiting GluN2A-NMDAR function following an excitotoxic insult with glutamate increases endogenous ROS generation and oxidative stress (88). Since glutamate leads to sequential activation of both GluN2A- and GluN2B-NMDARs and the detrimental effects glutamate are primarily mediated through GluN2B-NMDARs, the earlier findings from Zhu *et al.* imply that upregulation of GluN2A-NMDAR could protect neurons against glutamate-induced oxidative stress. Collectively, these findings further confirm that glutamate and homocysteine mediated NMDAR stimulation can have opposing effects on GluN2A-NMDAR and neuronal function. We further show that the sustained activation of ERK MAPK serves as the intermediary signaling pathway between GluN2A-NMDAR stimulation and mitochondrial ROS generation. Although ROS have been suggested to have an important role in sustaining ERK MAPK signaling, the role of ERK MAPK in regulating mitochondrial ROS generation in neurons has not been reported earlier. As such, these findings not only provide insight into the signaling pathways involved in homocysteine-induced sustained GluN2A-NMDAR stimulation but also demonstrate a new relationship between mitochondrial ROS generation and GluN2A-NMDAR activation.

## Experimental procedures

### Materials and reagents

Pregnant female Sprague-Dawley rats were purchased from Envigo. GluN2A-subunit KO mice (GluN2A KO) obtained from Dr Andrew Holmes, NIH/NIAAA (89), were bred and made time-pregnant at the animal facility of University of New Mexico. The Institutional Animal Care and Use Committee of University of New Mexico, HSC, approved all animal procedures. L-homocysteine thiolactone hydrochloride, glycine, EGTA, Hoechst 33342, DL-AP5 (DL-2-amino-5-phosphopentanoic acid), NVP-AAM077 ([(R)-[(S)-1-(4-bromo-phenyl)-ethylamino]-(2,3-dioxo-1,2,3,4-tetrahydro-quinoxalin-5-yl)-methyl]phosphonic acid), Ro 25-6981, PF431396, PP2, and mitoTEMPO ((2-(2,2,6,6-tetramethylpiperidin-1-oxyl-4-ylamino)-2-oxoethyl)triphenylphosphonium chloride) were purchased from Millipore Sigma. Fluo-3 acetooxymethyl ester (Fluo3-AM) and all cell culture reagents were purchased from Invitrogen. Anti-phospho-ERK1/2 (Thr202/Tyr204) monoclonal antibody (pERK), anti-ERK2 polyclonal antibody (ERK), anti-phospho-Pyk2 (Tyr402) polyclonal antibody, anti-phospho-SFK (Tyr416) polyclonal antibody (p-Src), anti-SFK monoclonal antibody, and anti-rabbit and anti-mouse horseradish peroxidase–conjugated secondary antibodies were purchased from Cell Signaling Technology. Anti-Pyk2 polyclonal antibody was obtained from Abcam. The 35-mm tissue culture dishes were obtained from MatTek Corporation and the poly-D-lysine-coated 60 mm dishes, and 4-well culture slides were obtained from Corning BD Biocoat.

### Neuron culture, L-homocysteine preparation, and stimulation

Embryos obtained from pregnant Sprague Dawley rats (16- to 17-day gestation), WT or GluN2A KO mice (15- to 16-day gestation) were used to establish primary cortical neuronal cultures as described earlier (20, 21, 90). For live-cell imaging involving assessment of Ca^2+^ and ROS levels, neurons were grown on glass-bottom 35 mm culture dishes coated overnight with poly-D-lysine (50 μg/ml) and Laminin (10 μg/ml). For biochemical studies, neurons were grown on poly-D-lysine-coated 60 mm dishes and for cell death assay, neurons were grown on poly-D-lysine-coated 4-well culture slides. Neuronal cultures were maintained in Modified Eagles Medium supplemented with 5% fetal bovine serum and antibiotic/antimycotic solution for 12 to 13 days. On the day of the experiment, the cells were treated with L-homocysteine (50 μM) in Hank’s balanced salt solution (HBSS) containing 50 μM of glycine as described previously (13, 20, 21, 90). L-homocysteine was prepared fresh each time from L-homocysteine thiolactone hydrochloride as previously described (20, 21). In some experiments, pharmacological inhibitors (DL-AP5, 200 μM; NVP-AAM077, 30 nM; Ro 25-6981, 5 μM; PF431396, 5 μM; PP2, 5 μM; or mitoTEMPO, 5 μM) were added 15 min prior to or 30 min after the onset of L-homocysteine treatment and maintained for the specified time periods. The cells were then subjected to either live-cell imaging for intracellular Ca^2+^ and ROS measurements or immunoblotting or cell death assay as described below.

### Calcium measurements

Intracellular Ca^2+^ in neurons was determined using the fluorescent indicator, Fluo3-AM. Neuronal cultures (12–13 days) from rat embryos and maintained in phenol red free HBSS were loaded with Fluo3-AM (4 μM, solubilized in dimethyl sulfoxide) for 30 min at 37 °C. Neurons were then maintained in HBSS only (30 min, 37 °C) to allow complete de-esterification of intracellular AM esters according to manufacturer’s protocol. Fluo3-AM loaded cells were treated with vehicle (saline) or L-homocysteine (50 μM) in the absence or presence of pharmacological inhibitors as specified in each experiment. Time lapse live-cell imaging was performed using a Nikon Ti Eclipse inverted microscope equipped with Tokai Hit stage top incubator maintained at 37 °C and infused with 95% air and 5% CO_2_ mixture. Fields of 5 to 8 cells were imaged using a 40× oil immersion objective (Nikon). Fluorescence excitations (488 nm) were provided with Sutter LB-LS/30 Lambda xenon arc lamp, and fluorescence emissions were captured using a charged-coupled device (CCD) camera from Photometrics. NIS Elements AR software (https://www.nisoftware.net/NikonSaleApplication/) was used for data acquisition and analysis. Data from regions of interest were corrected for background.

### Measurement of mitochondrial ROS

Primary neuronal cultures (8–9 days) from rat or mice embryos grown on glass-bottom 35-mm diameter culture dishes were transduced with adenovirus particles containing mito-RoGFP construct obtained from Viraquest Inc with permission from Dr Paul Schumacker, Northwestern University Feinberg School of Medicine (49). The RoGFP protein biosensor contains two engineered cysteine thiols created by introducing four mutations (C48S, Q80R, S147C, and Q204C) in the mammalian GFP expression vector (pEGFP-N1). The 48-bp region encoding the mitochondrial targeting sequence from cytochrome oxidase subunit IV was attached to the 5′ end of the coding sequence of RoGFP, followed by ligation into the VQ Ad5CMV K-NpA adenoviral shuttle vector to generate the mito-RoGFP construct (49). In normal conditions (reduced environment), the fluorescence intensity of the expressed mito-RoGFP is high at excitation maximum 484 nm, which decreases in the presence of ROS (oxidized environment) in the mitochondrial matrix (49). The adenoviral particles were transduced according to protocol suggested by Viraquest Inc. Briefly, 8 to 9 days old neuronal cultures were incubated with 2.5 μl of stock virus containing 1 × 10^9^ particles in 1 ml of original growth media and incubated for 16 to 18 h. Following incubation, the media containing the viral particles were removed and replaced with the original growth media. The cells were maintained in the culture media for another 4 to 5 days for expression of mito-RoGFP. For live-cell imaging, the culture media were replaced with HBSS media without phenol red and subjected to fluorescence imaging at 484 nm wavelength using a Nikon Ti Eclipse inverted microscope equipped with Tokai Hit stage top incubator maintained at 37 °C and infused with 95% air and 5% CO_2_ mixture. Baseline photomicrographs were acquired for 5 to 8 cells per field with 40× oil immersion objective and at 484 nm. L-Homocysteine (50 μM) or vehicle (saline) was added to the cells, and time-lapse microscopy was performed every 15 min for the first 30 min and every half hour thereafter. As positive control, a subset of mito-RoGFP–expressing neurons from rat cultures were treated with H_2_O_2_ (50 μM) and time-lapse microscopy was performed every 30 s for 10 min. In some studies, selective pharmacological inhibitors were added to the cells either 15 min prior to or 30 min after onset of L-homocysteine treatment and maintained for the duration of time-lapse microscopy, performed as described above. Relative fluorescent intensity was estimated using NIS Elements AS software and expressed as arbitrary units at 484 nm. The decrease in mito-RoGFP fluorescence intensity was taken as a measure of mitochondrial ROS generation and expressed as percent oxidized relative to baseline control.

### Immunoblotting

Primary neuronal cultures from rat and mice embryos were treated with or without L-homocysteine in the presence or absence of pharmacological inhibitors. The cells were harvested in SDS sample buffer (91). Equal protein from the cell lysates were resolved by SDS-PAGE (7.5%) and subjected to immunoblotting procedures as described earlier (20, 21, 43). The blots were analyzed with anti-pERK, anti-ERK, anti-pPyk2, anti-Pyk2, anti-pSrc family, and anti-Src family antibodies according to manufacturer’s protocol. Densitometric analyses of the images captured on X-ray films were performed using the Image J software (https://imagej.net/ij/).

### Hoechst DNA staining

Neuronal cultures obtained from rat embryos were treated with L-homocysteine for 18 h in the presence or absence of mitoTEMPO (5 μM), PF431396 (5 μM), PP2 (5 μM) DL-AP5 (200 μM), NVP-AAM077 (30 nM), PD98059 (15 μM), or Ro 25-6951 (5 μM). Cells were fixed with 4% paraformaldehyde and incubated with Hoechst 33342 dye as described earlier (20). Percentage of pyknotic nuclei was quantitatively assessed by fluorescent microscopy to determine the extent of neuronal death. A total of 1500 cells were counted from at least 15 fields for each experimental condition (n = 4).

### Statistical analysis

Statistical analyses were performed using GraphPad Prism software (version 10.2.1; https://www.graphpad.com). Immunoblot data evaluating the effects of drug treatment on protein phosphorylation at a given time point were analyzed by computer assisted densitometry and Image J analysis. Quantitative effects of drug treatment were assessed by one-way ANOVA, followed by Bonferroni’s multiple comparison test. For evaluating the effects of drug treatment on changes in intracellular Ca^2+^ level and mitochondrial ROS generation over time, data were analyzed using two-way repeated measures ANOVA with drug treatment and time as the two factors, followed by Bonferroni’s post hoc test for detailed group comparisons. Effects of drug treatment on cell death were assessed by one-way ANOVA, followed by Bonferroni’s multiple comparison test. Greenhouse-Geisser correction was applied, where test of sphericity failed. Data are expressed as mean ± SD and significance was set at *p* <0.05.

## Data availability

All data are contained within the article.

## Conflict of interest

The authors declare that they have no conflicts of interest with the contents of this article.
